# The complete mitochondrial genome of the Ferruginous Duck (*Aythya nyroca*) from Ningxia, China

**DOI:** 10.1080/23802359.2020.1870901

**Published:** 2021-02-12

**Authors:** Hao Zhai, Zongzhi Li, Shuhui Mi, Dehuai Meng, Hongxian Yu, Liwei Teng, Zhensheng Liu

**Affiliations:** a College of Wildlife And Protected Area, Northeast Forestry University, Harbin, China; b Key Laboratory of Conservation Biology, National Forestry And Grassland Administration, Harbin, China

**Keywords:** Complete mitochondrial genome, *Aythya nyroca*, duck

## Abstract

The Ferruginous Duck (*Aythya nyroca*) is a diving duck that is widely distributed in Asia, Africa, and Europe. We determined the complete mitogenome of the Ferruginous Duck gathered at Ningxia, China. The total length of the complete mitochondrial genome is 16,623 bp and it consists of 13 protein-coding, 22 tRNA, 2 rRNA genes, and 1 control region (CR). Only one overlap among the 13 protein-coding genes was found: ND4L/ND4. The CR is 1068 bp in length. The nucleotide composition is 29.66% A, 22.28% T, 15.35% G, 32.71% C. The result of phylogenetic analysis showed that there is close genetic relationship among *Aythya nyroca* and three ducks in the Genus *Aythya*.

The Ferruginous Duck (*Aythya nyroca*), also named white-eyed pochard, is a partial migrant, medium-sized diving bird with a range spanning Europe, Asia, and Africa (Robinson and Hughes 2006). The Duck prefers shallow fresh waterbodies with rich submerged and floating vegetation with dense stands of emergent vegetation on the margins (Snow and Perrins 1998). According to the Red List of Threatened Species, this species is listed as Near Threatened, and destruction of the duck’s favored habitats across its range is thought to be the primary cause for its disjunct distribution and declining numbers (BirdLife International 2019). Also, the introduction of non-native species and climate change may pose a threat to the duck.

The complete mitochondrial genome of the Ferruginous Duck was sequenced using muscle tissue collected from Ningxia, China (105°57′E, 37°44′N), and the specimen was deposited in College of Wildlife and Protected Area, Northeast Forestry University (No.BYQY201004). DNA library was constructed using MGIEasy DNA Library Prep Kit (MGI, China) and sequenced by MGI MGISEQ-2000 with 150 paired-ends. The annotation and phylogenetic tree of the mitogenome sequence were conducted by MITOS (Bernt et al. 2013) and MEGA 7 (Kumar et al. 2018), respectively. The sequence was submitted to the GenBank with the accession number MW287344 and the raw sequencing data were deposited in SRA with the accession number PRJNA685540.

The mitochondrial genome consists of 13 protein-coding genes, 2 rRNA genes (12S rRNA and 16S rRNA), 22 tRNA genes, and 1 control region (CR). The total length of the genome is 16,623 bp, with a base composition of 29.66% A, 22.28% T, 15.35% G, and 32.71% C. The total length of 13 protein-coding genes is 10,997 bp long, all of which are encoded on the same strand except for ND6 in the light strand. Except for ND2, ND5 and Cytb (ATC start codon), ND6 (TAA start codon), and COX1,COX2 (GTG start codon), the remaining 7 protein-coding genes initiate with ATG (COX3, ND1, ND3, ND4, ND4L, ATP6, ATP8). The total length of all tRNA genes is 1544 bp long, and ranging from 66 bp (tRNA-Ser and tRNA-Cys) to 76 bp (tRNA-Trp). Lengths of two rRNA genes and control region are 984 bp (12 s rRNA), 1604 bp (16S rRNA), and 1068 bp (control region), respectively.

The previously reported 14 species in family Anatidae and *Aythya nyroca* mitogenome sequence were aligned using ClustalW and phylogenetic tree was constructed by the neighbor-joining method (Saitou and Nei 1987) and conducted in MEGA7 (Kumar et al. 2016). The bootstrap consensus tree inferred from 1000 replicates is taken to represent the evolutionary history of the taxa analyzed (Felsenstein 1985) (Figure 1). The phylogenetic tree indicated that the phylogenetic relationship of Ferruginous Duck is very close to the three species in the Genus *Aythya*: *Aythya ferina*, *Aythya americana* and *Aythya fuligula*.

**Figure 1.**
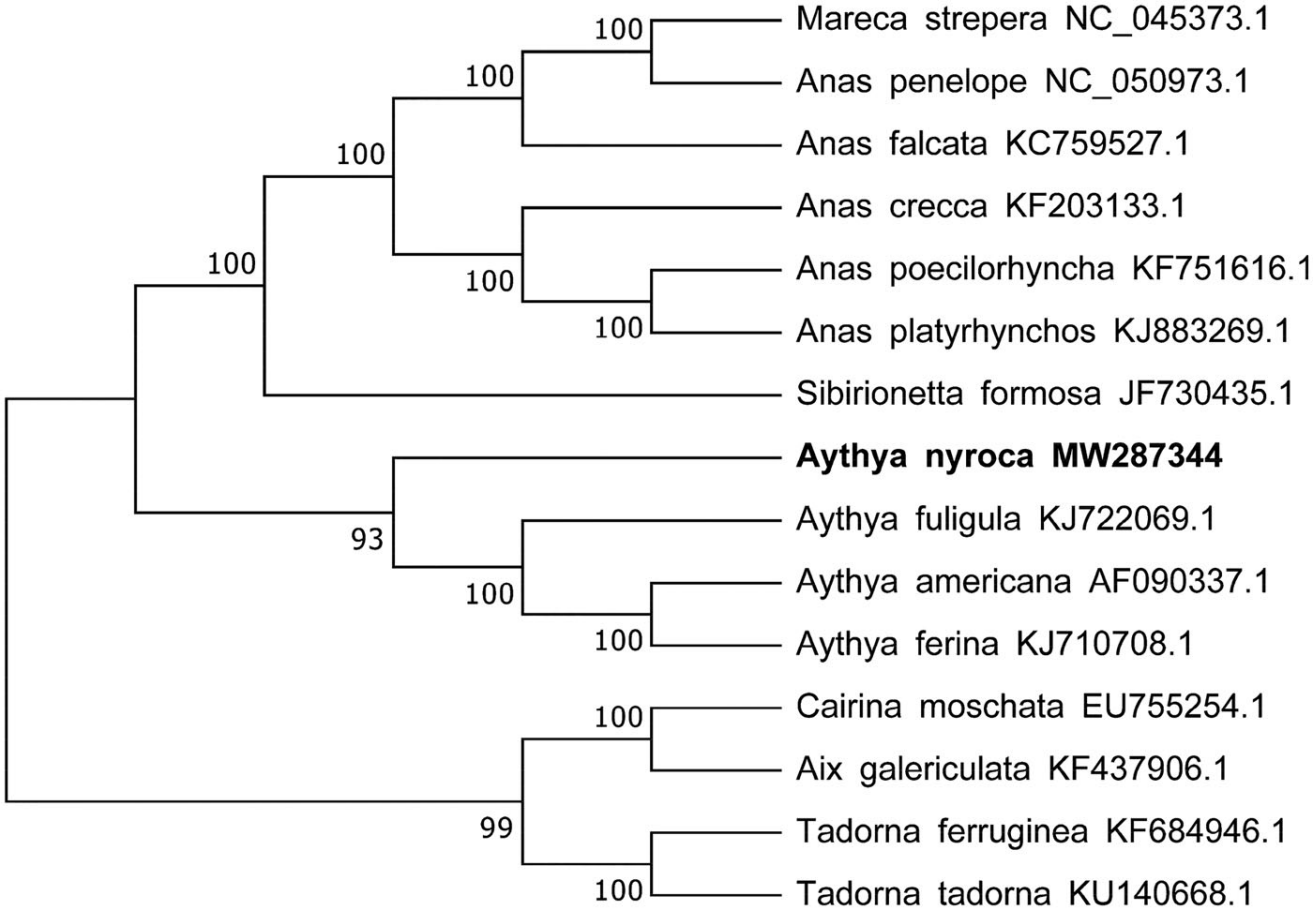
Phylogenetic tree generated using the neighbor-joining method based on complete mitochondrial genomes of 14 species in Anseriformes: Anatidae.

## Data Availability

The genome sequence data that support the findings of this study are openly available in GenBank of NCBI at (https://www.ncbi.nlm.nih.gov/) under the accession no. MW287344. The associated SRA number is PRJNA685540.

## References

[CIT0001] Bernt M, Donath A, Jühling F, Externbrink F, Florentz C, Fritzsch G, Pütz J, Middendorf M, Stadler PF. 2013. MITOS: improved de novo metazoan mitochondrial genome annotation. Mol Phylogenet Evol. 69(2):313–319. 22982435 10.1016/j.ympev.2012.08.023

[CIT0002] BirdLife International. 2019. *Aythya nyroca*. The IUCN Red List of Threatened Species 2019: e.T22680373A152620862.

[CIT0003] Felsenstein J. 1985. Confidence limits on phylogenies: an approach using the bootstrap. Evolution. 39(4):783–791. 28561359 10.1111/j.1558-5646.1985.tb00420.x

[CIT0004] Kumar S, Stecher G, Tamura K. 2016. MEGA7: molecular evolutionary genetics analysis version 7.0 for bigger datasets. Mol Biol Evol. 33(7):1870–1874. 27004904 10.1093/molbev/msw054 PMC8210823

[CIT0005] Robinson JA, Hughes B. 2006. International single species action plan for the conservation of the Ferruginous Duck *Aythya nyroca*. Bonn: Convention on the Conservation of Migratory Species of Wild Animals/Agreement on the Conservation of African Eurasian Migratory Waterbirds.

[CIT0006] Saitou N, Nei M. 1987. The neighbor-joining method: a new method for reconstructing phylogenetic trees. Mol Biol Evol. 4(4):406–425. 3447015 10.1093/oxfordjournals.molbev.a040454

[CIT0007] Snow DW, Perrins CM. 1998. The birds of the Western Palearctic Concise edition volume 1 non-passerines. London: Oxford University Press.

